# Supplementary material for: In Silico Substrate-Binding Profiling for SARS-CoV-2 Main Protease (Mpro) Using Hexapeptide Substrates
Source: Viruses. 2023 Jun 29;15(7):1480. doi: 10.3390/v15071480 (PMC10385622; doi:10.3390/v15071480)
Supplement: Supplementary file 1 [file viruses-15-01480-s001.zip › viruses-2451087-supplementary.pdf]

In Silico Substrate-Binding Profiling for SARS-CoV-2 Main Protease (M^{pro}) Using Hexapeptide Substrates

Sophakama Zabo and Kevin Alan Lobb *

Supplementary Material

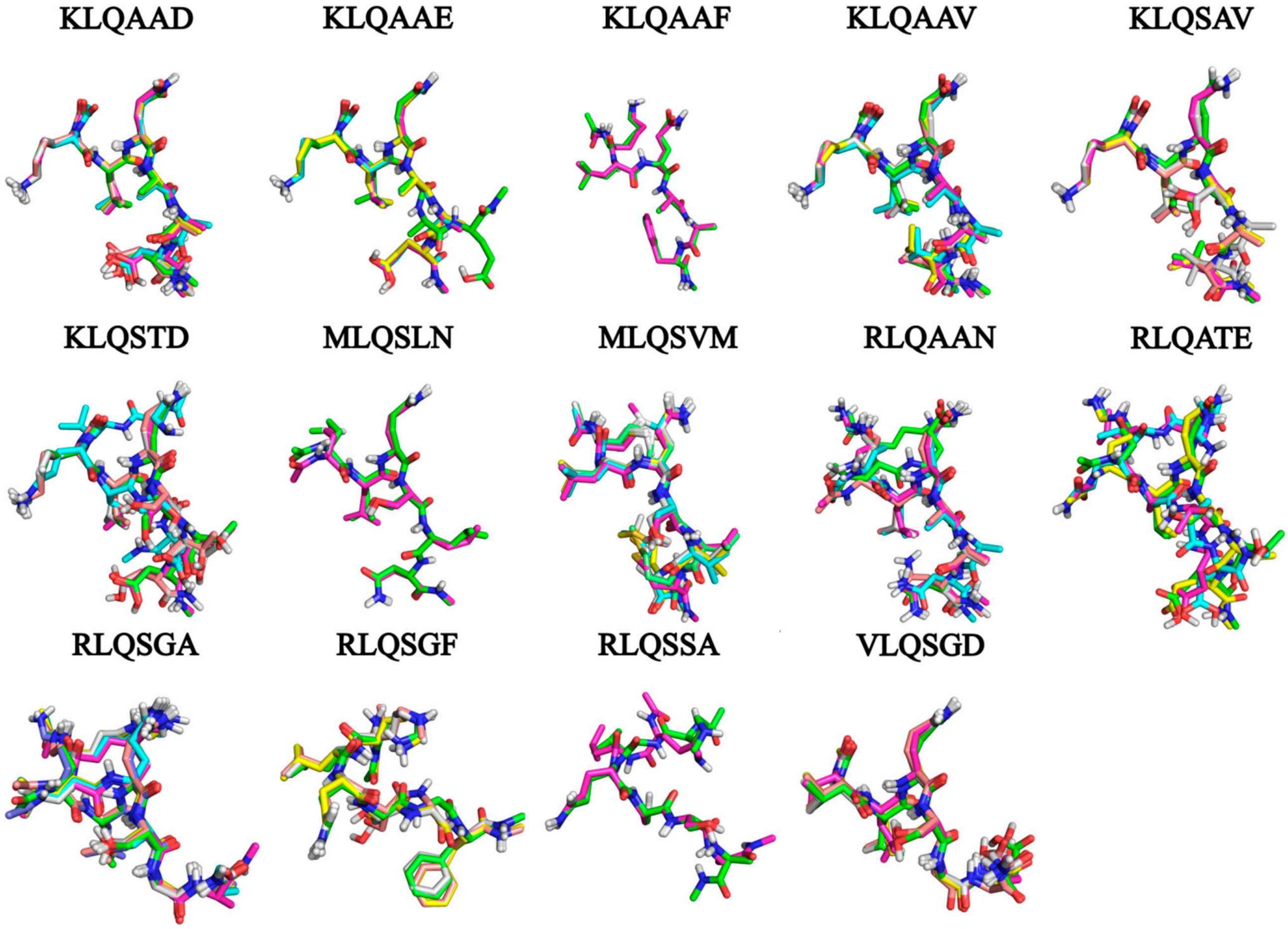

Supplementary Figure S1. Validation of reproducibility of the best poses in the docking results. The visualisation of the best poses of substrates with all 100 conformers docked. Image was generated using PyMOL.

Supplementary Table S1: The ligand efficiencies of the hexapeptide substrates docked onto SARS-CoV-2 M^{pro} on basis of recognition sequence.

Sequence	Docking Score (kcal.mol^{-1})	HA	LE (kcal.mol^{-1} per atom)
LQ↓A	-8.0 ± 0.28	22	-0.4 ± 0.01
LQ↓S	-7.9 ± 0.28	23	-0.3 ± 0.01

HA: heavy atoms (non-hydrogen atoms); LE: ligand efficiency.

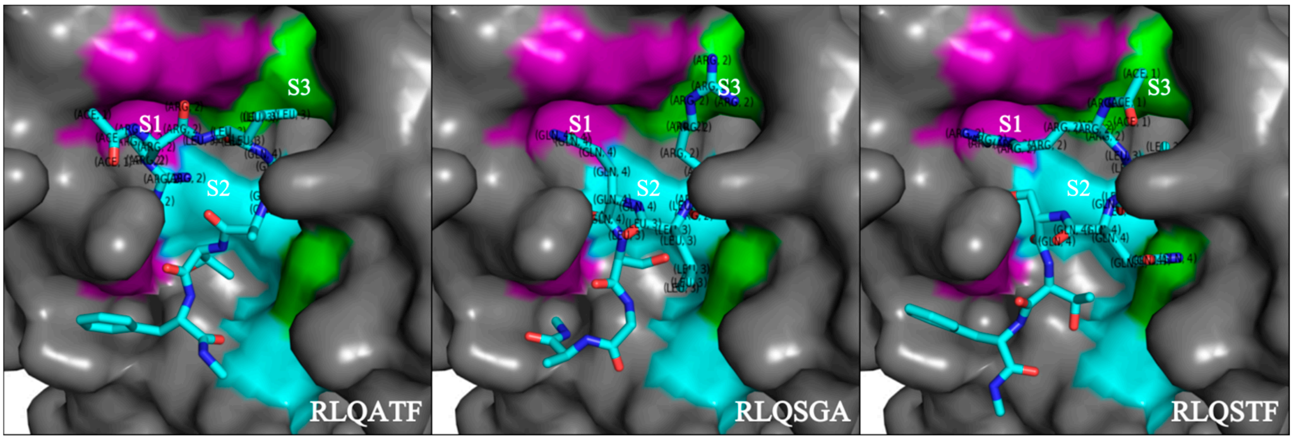

Supplementary Figure S2. Confirmation of SARS-CoV-2 M^{pro} substrate recognition in binding poses for substrates RLQATF, RLQSGA and RLQSTF. The surface of SARS-CoV-2 M^{pro} (PDB ID:6XHM) showing docked substrates and substrate binding subsites colour-coded as follows: Purple: S1, Cyan: S2; Green: S3. The substrates attained a docking score of -8.7 kcal.mol^{-1}. The image was generated using PyMOL.

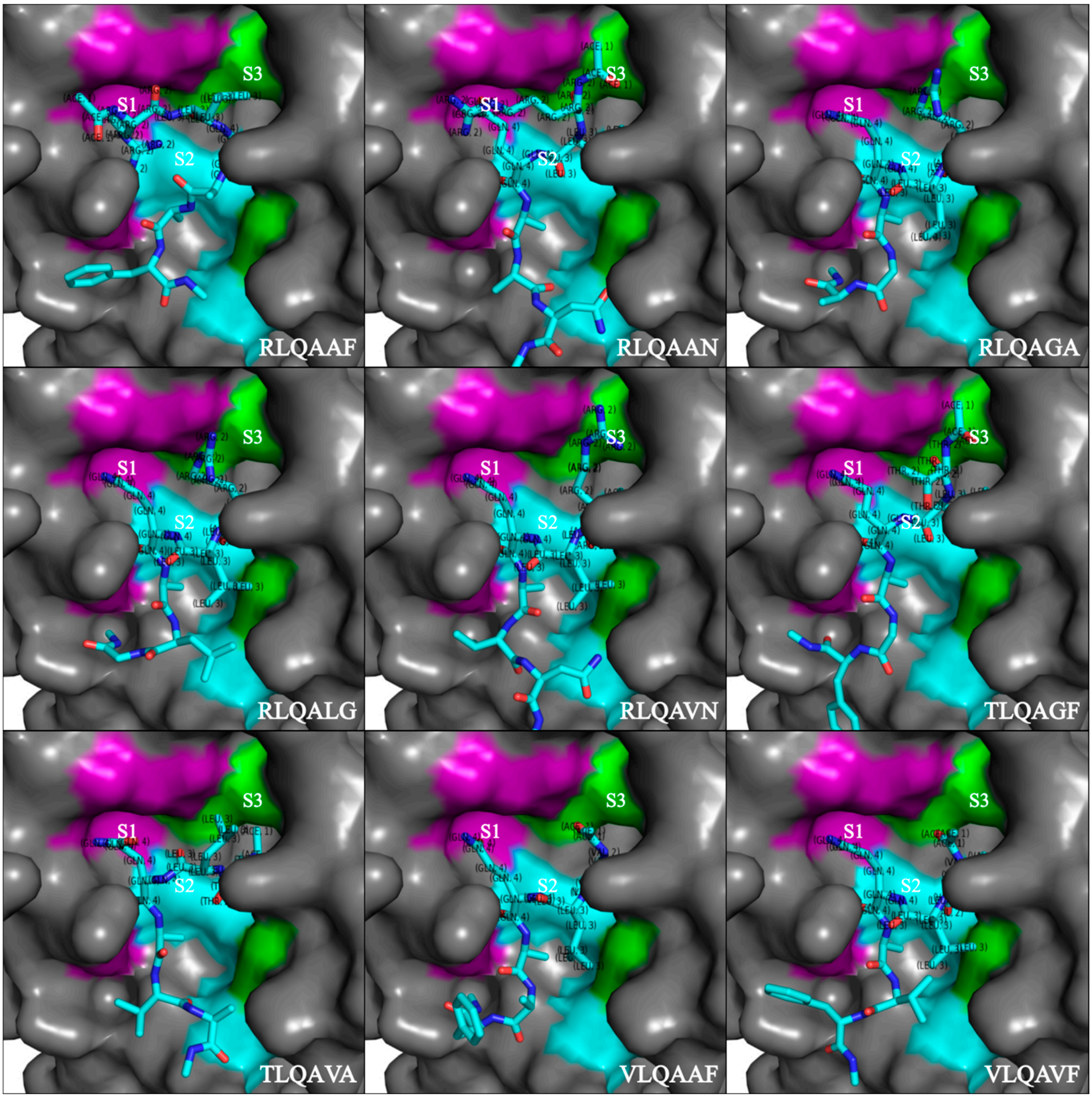

Supplementary Figure S3. Confirmation of SARS-CoV-2 M^{pro} substrate recognition in binding poses for substrates RLQAAN, RLQAAF, RLQAGA, RLQALG, RLQAVN, TLQAGF, TLQAVA, VLQAAF and VLQAVF. The surface of SARS-CoV-2 M^{pro} (PDB ID:6XHM) showing docked substrates and substrate binding subsites colour-coded as follows: Purple: S1, Cyan: S2; Green: S3. The substrates attained a docking score of -8.6 kcal.mol^{-1}. The image was generated using PyMOL.

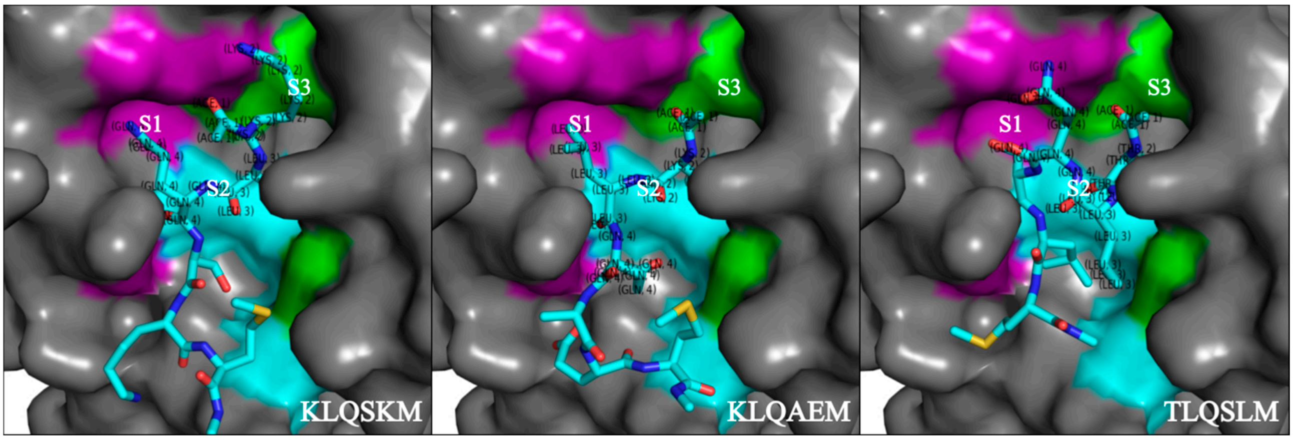

Supplementary Figure S4. Confirmation of SARS-CoV-2 M^pro^ substrate recognition in binding poses for substrates KLQSKM, KLQAEM and TLQSLM. The surface of SARS-CoV-2 M^pro^ (PDB ID:6XHM) showing docked substrates and substrate binding subsites color-coded as follows: Purple: S1, Cyan: S2; Green: S3. The substrates attained docking score of -7.0 kcal.mol^{-1} (KLQSKM) and -7.1 kcal.mol^{-1} (KLQAEM and TLQSLM). Image was generated using PyMOL

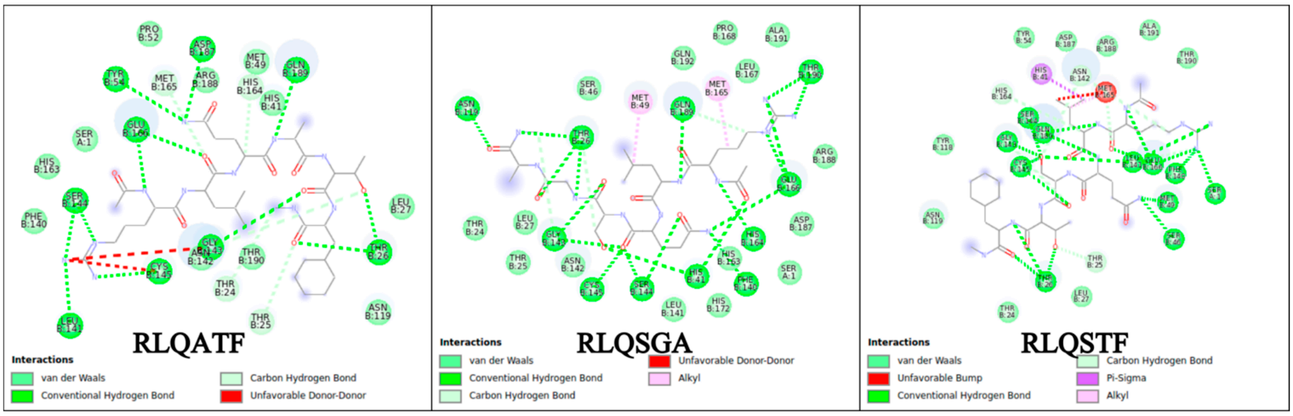

Supplementary Figure S5. Resolution of intermolecular interactions between M^{pro} and substrates at the active site. 2D representation of the protein-ligand interactions at active sites for M^{pro} complexed with RLQATF, RLQSGA and RLQSTF hexapeptides. The substrates attained a docking score of -8.6 kcal.mol^{-1}. The images were generated on BIOVIA Discovery Studio 2020 Client.

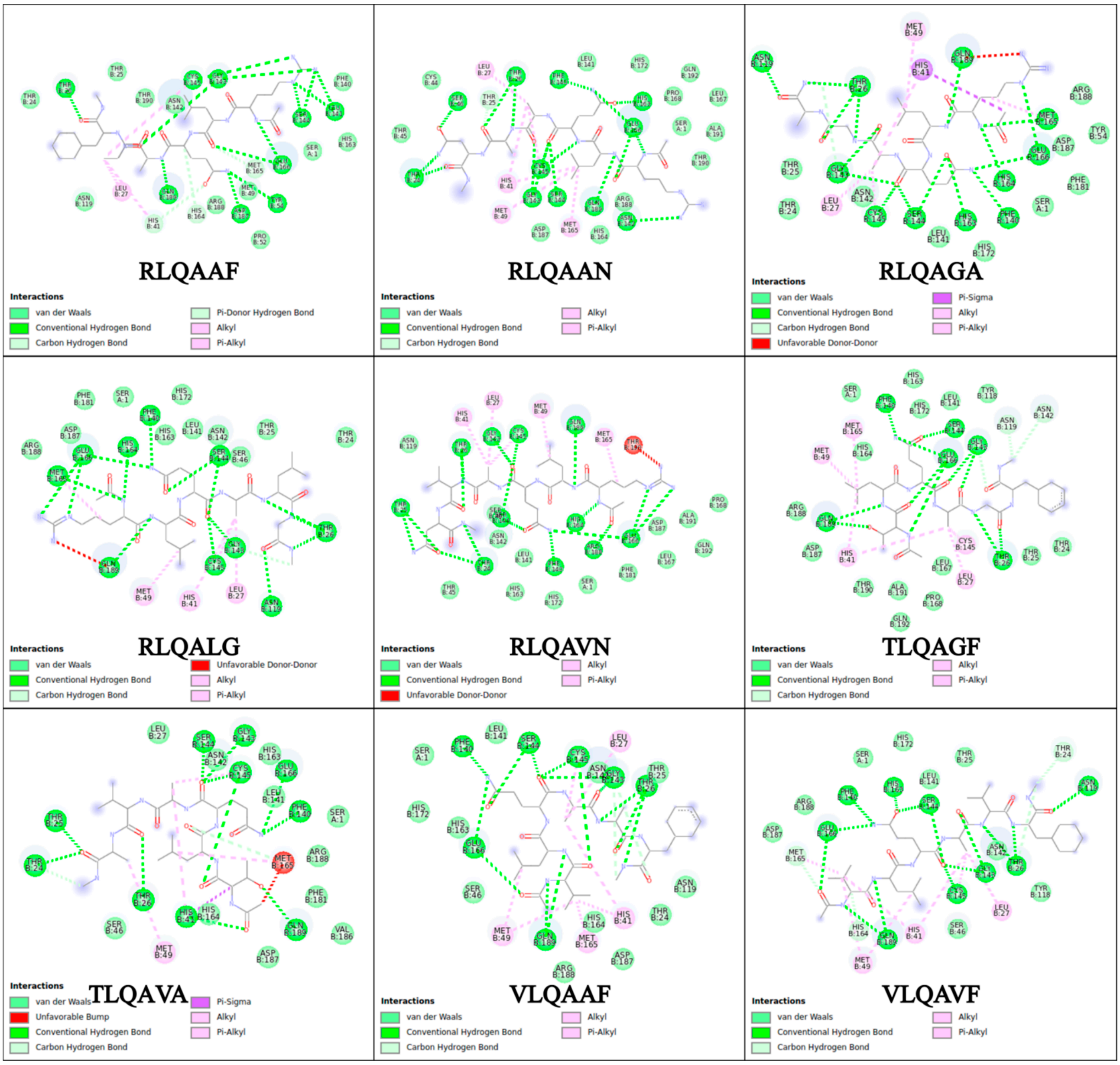

Supplementary Figure S6. Resolution of intermolecular interactions between M^{pro} and substrates at the active site. 2D representation of the protein-ligand interactions at active sites for M^{pro} complexed with RLQAAF, RLQAAN, RLQAGA, RLQALG, RLQAVN, TLQAGF, TLQAVA, VLQAAF and VLQAVF hexapeptides. The substrates attained a docking score of -8.6 kcal.mol^{-1}. The images were generated on BIOVIA Discovery Studio 2020 Client.

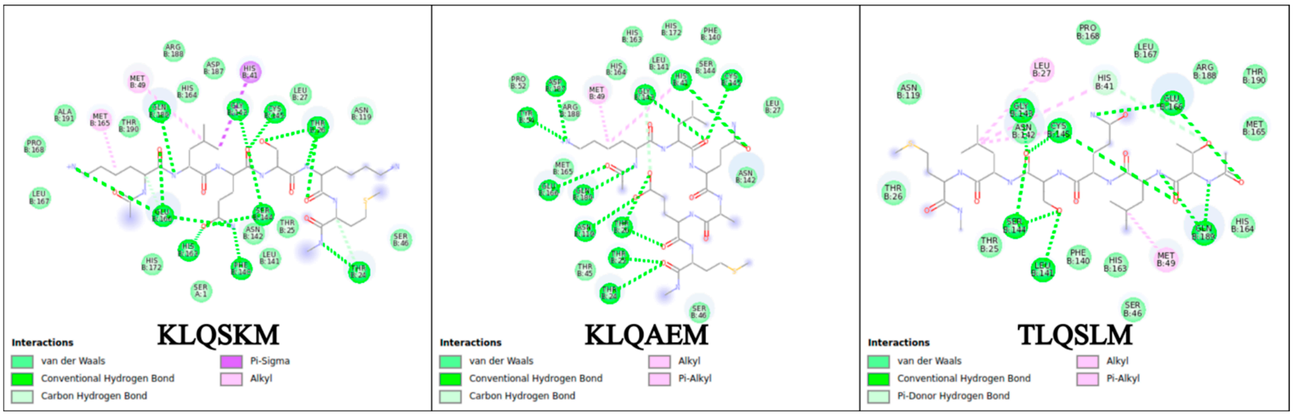

Supplementary Figure S7. Resolution of intermolecular interactions between M^{pro} and substrates at the active site. 2D representation of the protein-ligand interactions at active sites for M^{pro}

complexed with KLQSKM, KLQAEM and TLQSLM hexapeptides. The substrates attained docking score of -7.0 kcal.mol^{-1} (KLQSKM) and -7.1 kcal.mol^{-1} (KLQAEM and TLQSLM). The images were generated on BIOVIA Discovery Studio 2020 Client.

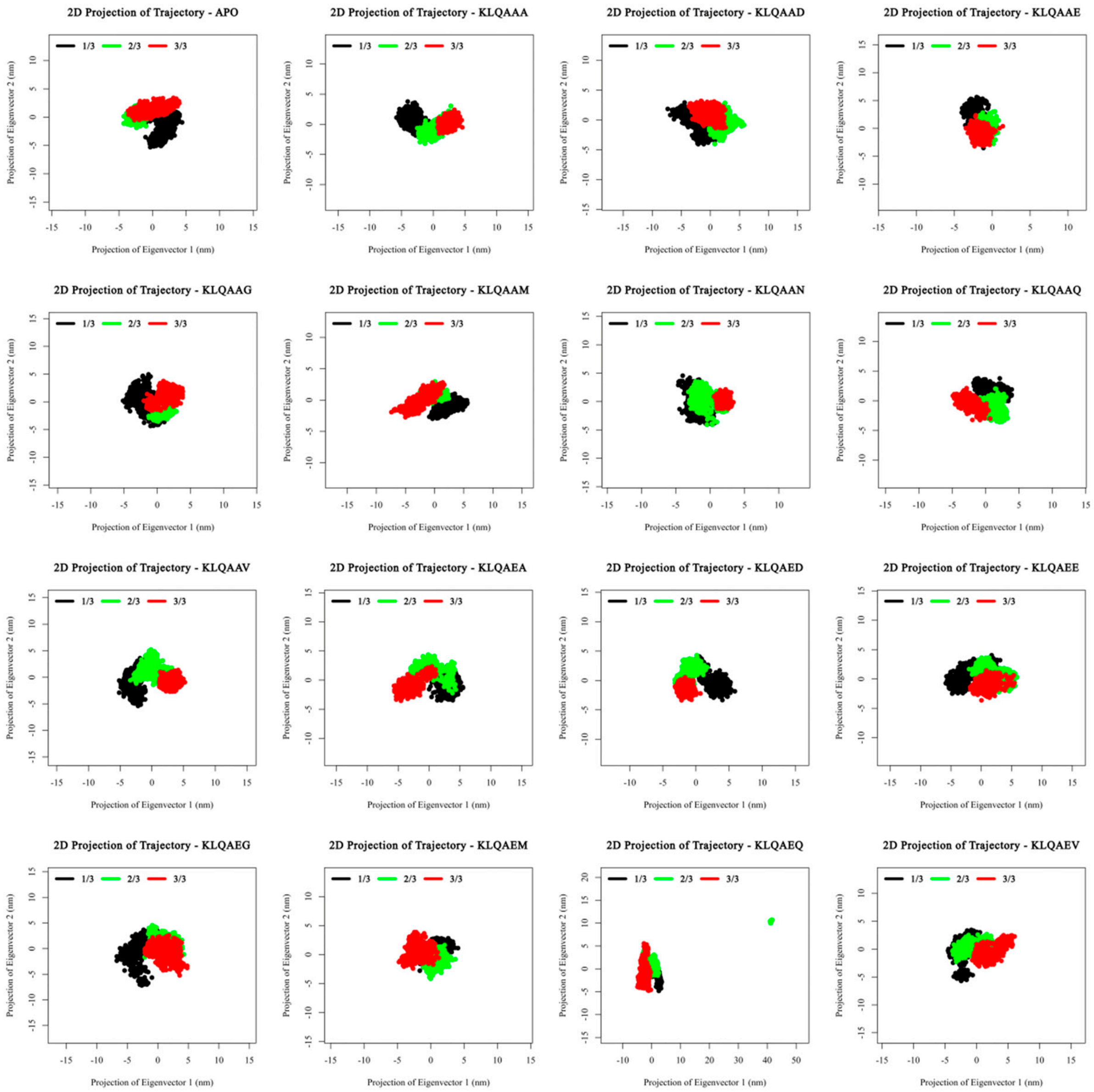

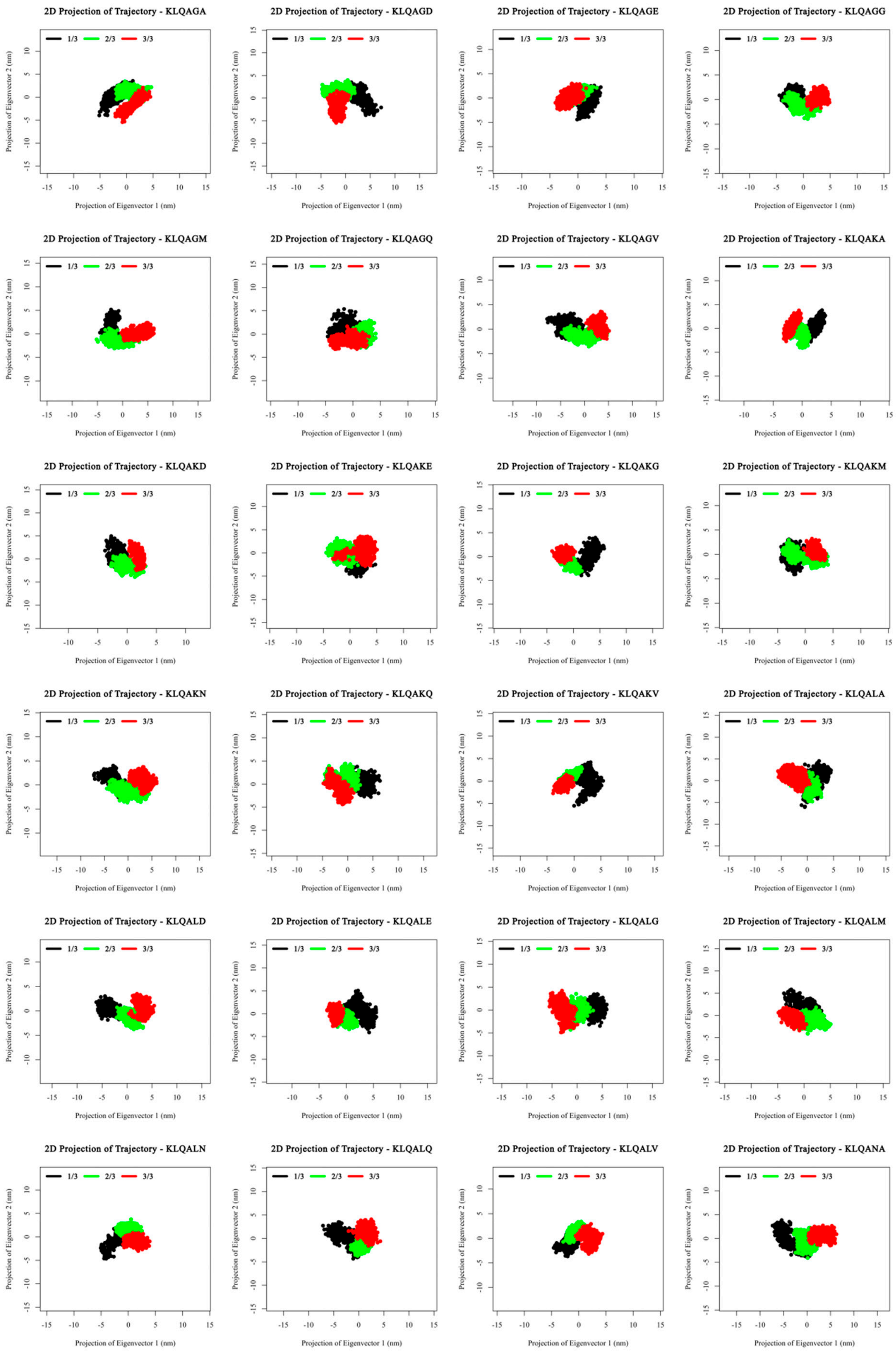
2D Projection of Trajectory - KLQAGA
2D Projection of Trajectory - KLQAGD
2D Projection of Trajectory - KLQAGE
2D Projection of Trajectory - KLQAGG
2D Projection of Trajectory - KLQAGM
2D Projection of Trajectory - KLQAGQ
2D Projection of Trajectory - KLQAGV
2D Projection of Trajectory - KLQAKA
2D Projection of Trajectory - KLQAKD
2D Projection of Trajectory - KLQAKE
2D Projection of Trajectory - KLQAKG
2D Projection of Trajectory - KLQAKM
2D Projection of Trajectory - KLQAKN
2D Projection of Trajectory - KLQAKQ
2D Projection of Trajectory - KLQAKV
2D Projection of Trajectory - KLQALA
2D Projection of Trajectory - KLQALD
2D Projection of Trajectory - KLQALE
2D Projection of Trajectory - KLQALG
2D Projection of Trajectory - KLQALM
2D Projection of Trajectory - KLQALN
2D Projection of Trajectory - KLQALQ
2D Projection of Trajectory - KLQALV
2D Projection of Trajectory - KLQANA
1/3
2/3
3/3
Projection of Eigenvector 1 (nm)
Projection of Eigenvector 2 (nm)

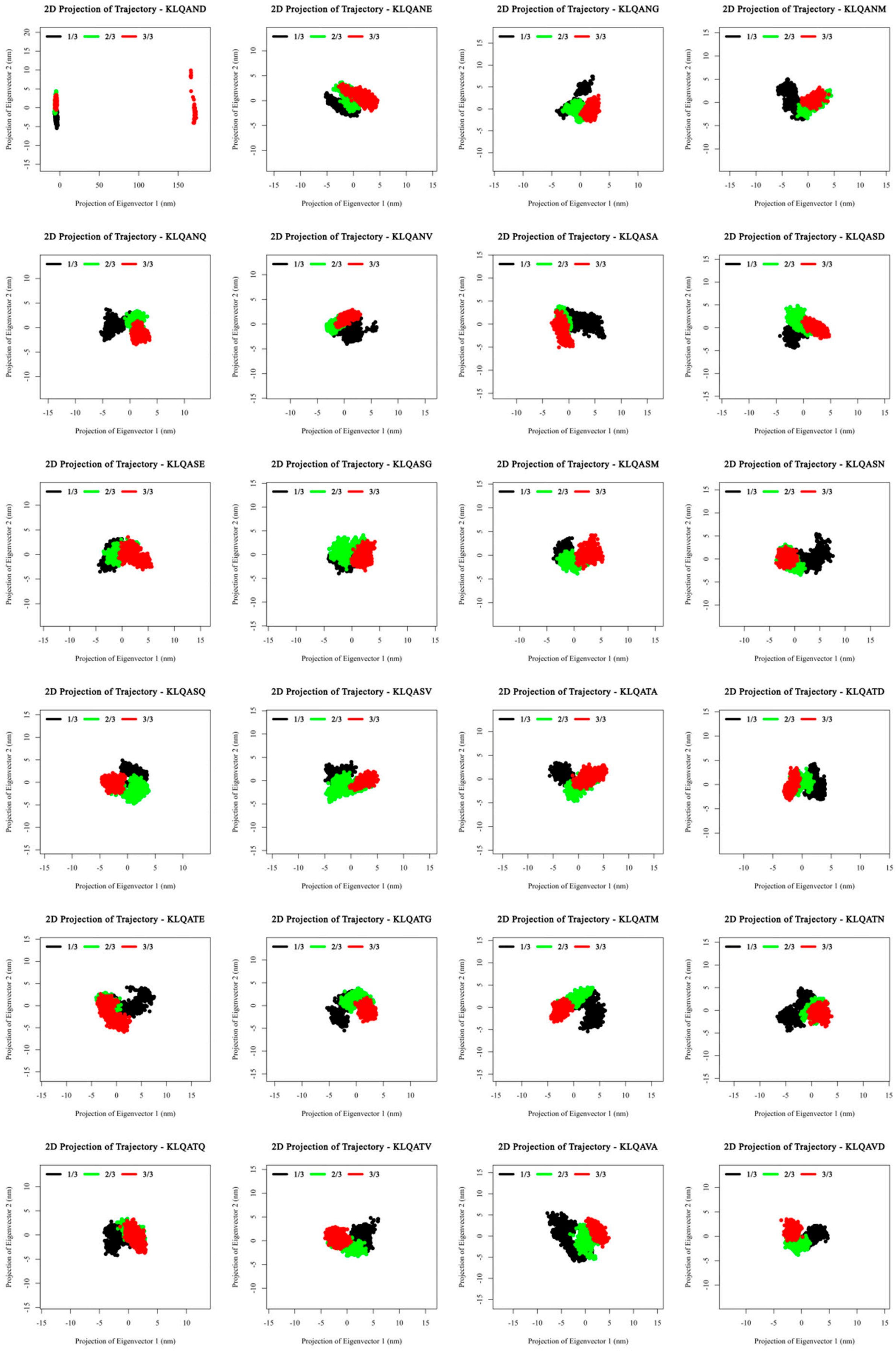
2D Projection of Trajectory - KLQAND
2D Projection of Trajectory - KLQANE
2D Projection of Trajectory - KLQANG
2D Projection of Trajectory - KLQANM
2D Projection of Trajectory - KLQANQ
2D Projection of Trajectory - KLQANV
2D Projection of Trajectory - KLQASA
2D Projection of Trajectory - KLQASD
2D Projection of Trajectory - KLQASE
2D Projection of Trajectory - KLQASG
2D Projection of Trajectory - KLQASM
2D Projection of Trajectory - KLQASN
2D Projection of Trajectory - KLQASQ
2D Projection of Trajectory - KLQASV
2D Projection of Trajectory - KLQATA
2D Projection of Trajectory - KLQATD
2D Projection of Trajectory - KLQATE
2D Projection of Trajectory - KLQATG
2D Projection of Trajectory - KLQATM
2D Projection of Trajectory - KLQATN
2D Projection of Trajectory - KLQATQ
2D Projection of Trajectory - KLQATV
2D Projection of Trajectory - KLQAVA
2D Projection of Trajectory - KLQAVD
1/3
2/3
3/3
Projection of Eigenvector 1 (nm)
Projection of Eigenvector 2 (nm)

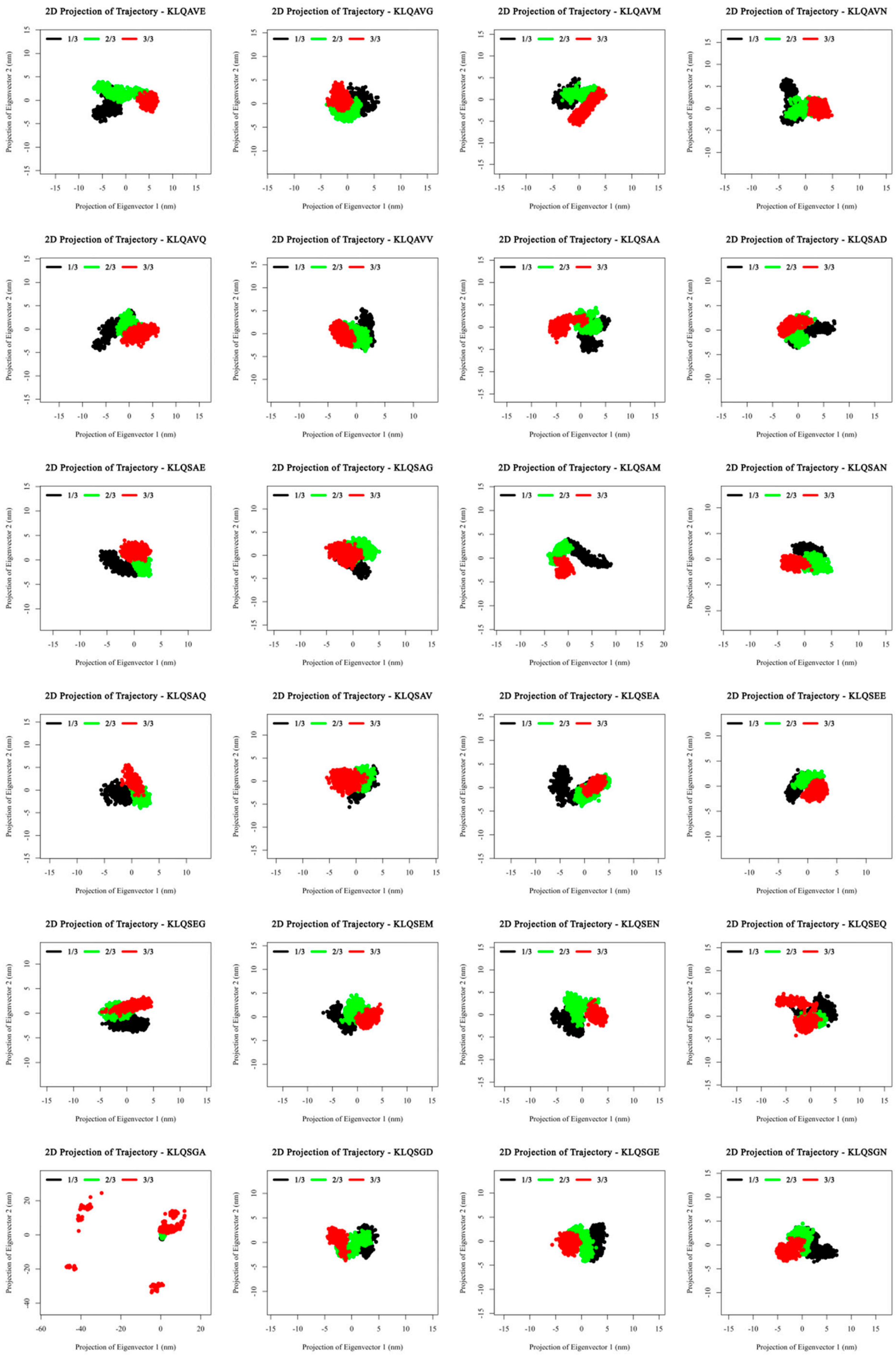
2D Projection of Trajectory - KLQAVE
2D Projection of Trajectory - KLQAVG
2D Projection of Trajectory - KLQAVM
2D Projection of Trajectory - KLQAVN
2D Projection of Trajectory - KLQAVQ
2D Projection of Trajectory - KLQAVV
2D Projection of Trajectory - KLQSAA
2D Projection of Trajectory - KLQSAD
2D Projection of Trajectory - KLQSAE
2D Projection of Trajectory - KLQSAG
2D Projection of Trajectory - KLQSAM
2D Projection of Trajectory - KLQSAN
2D Projection of Trajectory - KLQSAQ
2D Projection of Trajectory - KLQSAV
2D Projection of Trajectory - KLQSEA
2D Projection of Trajectory - KLQSEE
2D Projection of Trajectory - KLQSEG
2D Projection of Trajectory - KLQSEM
2D Projection of Trajectory - KLQSEN
2D Projection of Trajectory - KLQSEQ
2D Projection of Trajectory - KLQSGA
2D Projection of Trajectory - KLQSGD
2D Projection of Trajectory - KLQSGE
2D Projection of Trajectory - KLQSGN
1/3
2/3
3/3
Projection of Eigenvector 1 (nm)
Projection of Eigenvector 2 (nm)

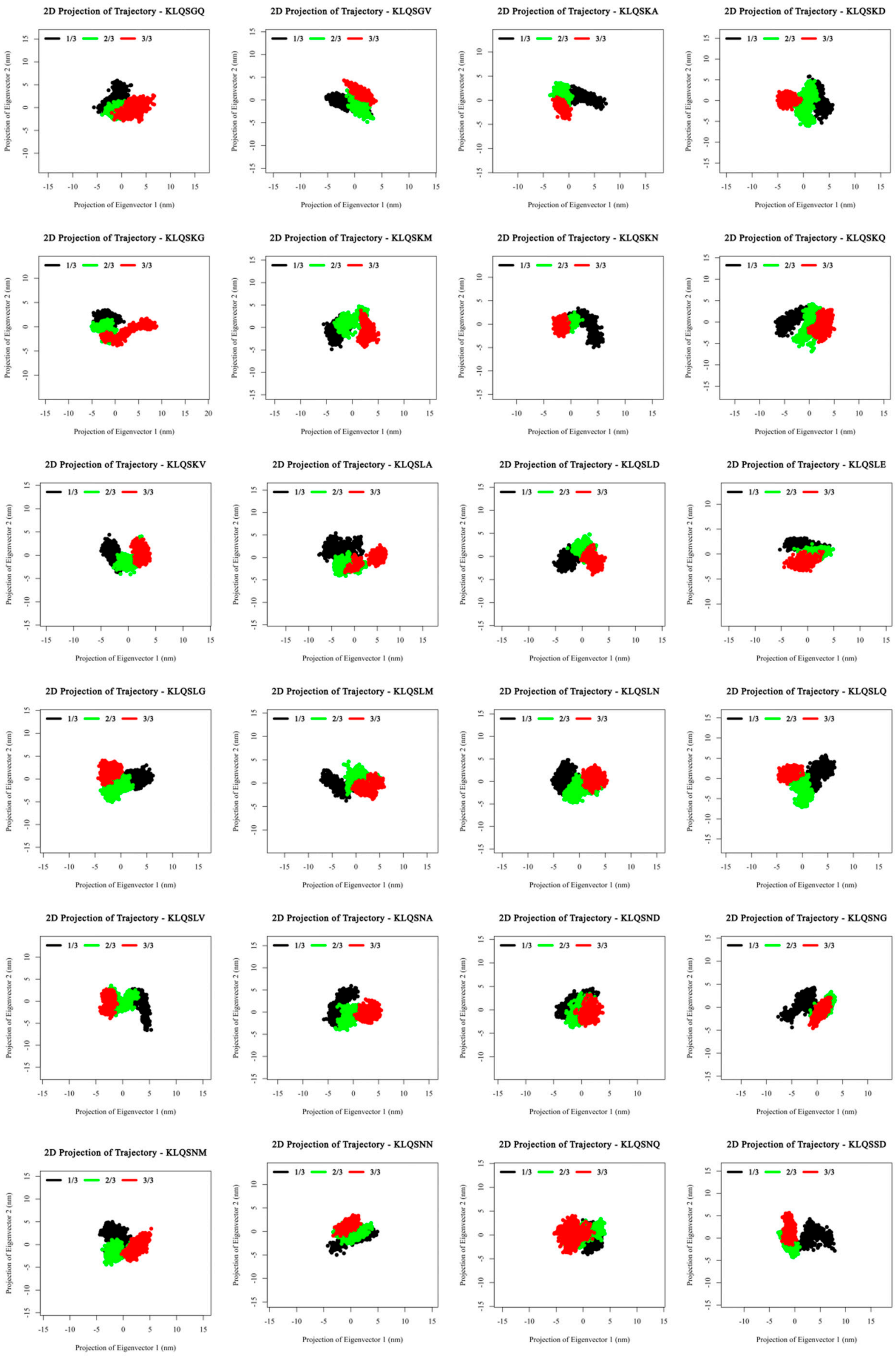
2D Projection of Trajectory - KLQSGQ
2D Projection of Trajectory - KLQSGV
2D Projection of Trajectory - KLQSKA
2D Projection of Trajectory - KLQSKD
2D Projection of Trajectory - KLQSKG
2D Projection of Trajectory - KLQSKM
2D Projection of Trajectory - KLQSKN
2D Projection of Trajectory - KLQSKQ
2D Projection of Trajectory - KLQSKV
2D Projection of Trajectory - KLQSLA
2D Projection of Trajectory - KLQSLD
2D Projection of Trajectory - KLQSLE
2D Projection of Trajectory - KLQSLG
2D Projection of Trajectory - KLQSLM
2D Projection of Trajectory - KLQSLN
2D Projection of Trajectory - KLQSLQ
2D Projection of Trajectory - KLQSLV
2D Projection of Trajectory - KLQSNA
2D Projection of Trajectory - KLQSND
2D Projection of Trajectory - KLQSNG
2D Projection of Trajectory - KLQSNM
2D Projection of Trajectory - KLQSNN
2D Projection of Trajectory - KLQSNQ
2D Projection of Trajectory - KLQSSD
1/3
2/3
3/3
Projection of Eigenvector 1 (nm)
Projection of Eigenvector 2 (nm)

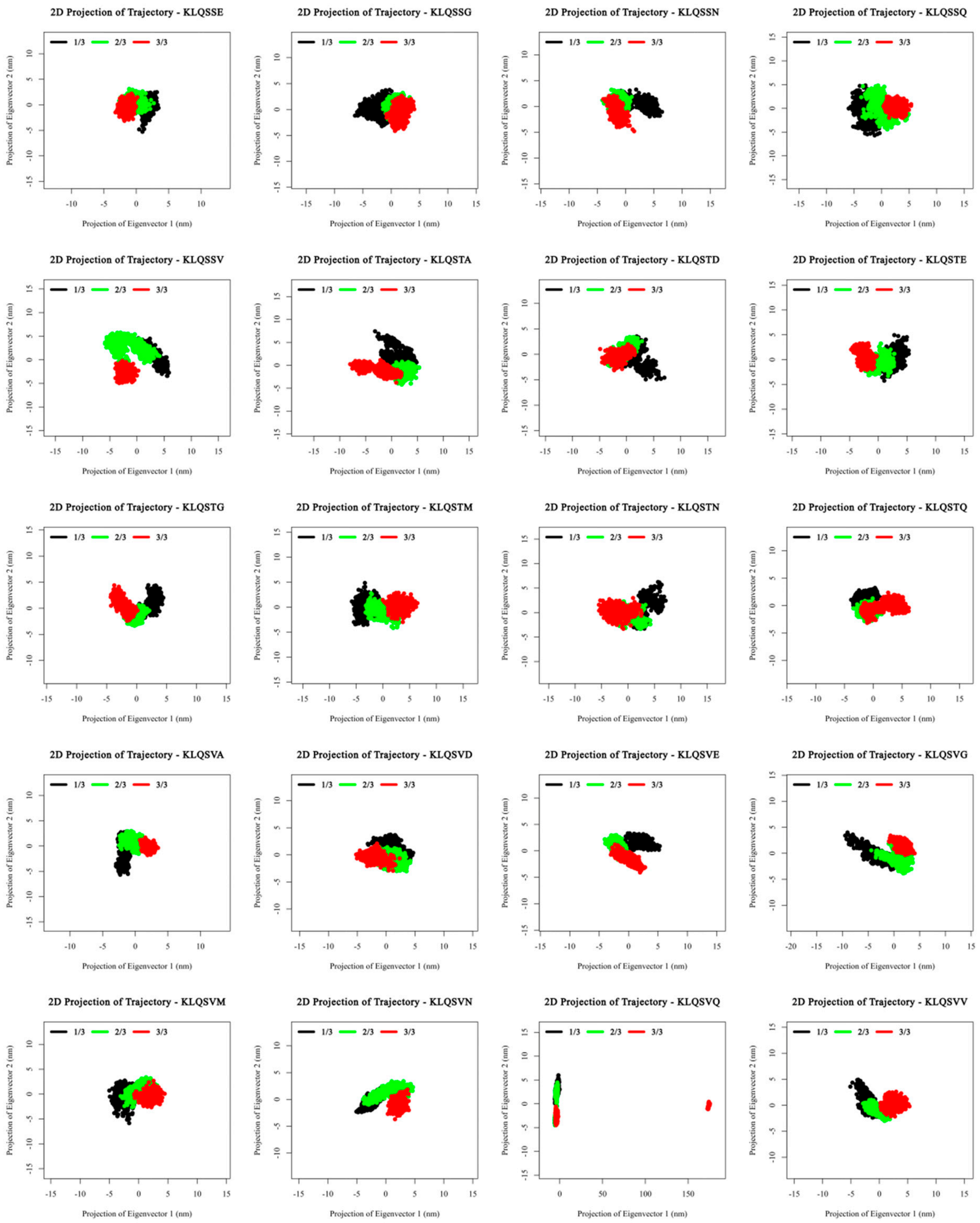

Supplementary Figure S8. The 2D projections of the principal components for M^{pro} (*apo* and KLQ***-substrate-bound) systems over the duration of the 20 ns MD simulations. The projection of the motion along phase space for PC1 and PC2 of M^{pro} *apo* and KLQ***-substrate-bound systems, showing the first third (black), second third (green) and final third (red) of the 20 ns simulation. Images were generated using Xmgrace (of Grace 5) and RStudio.

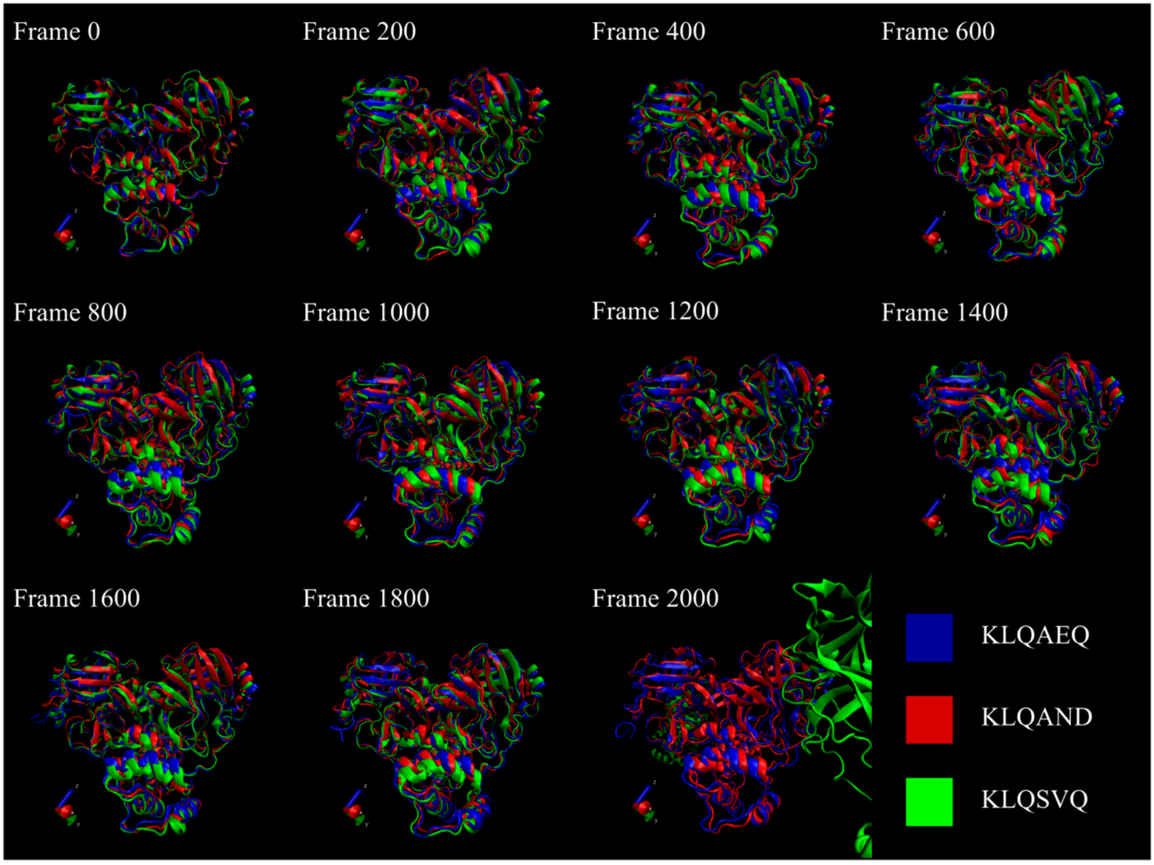

Supplementary Figure S9. Visualisation of the MD trajectories for Group 1 systems. Protein systems are shown cartoon representation, showing M^{pro}-KLQAEQ in blue; M^{pro}-KLQSVQ in red and M^{pro}-KLQAND in green. Images were generated using VMD.

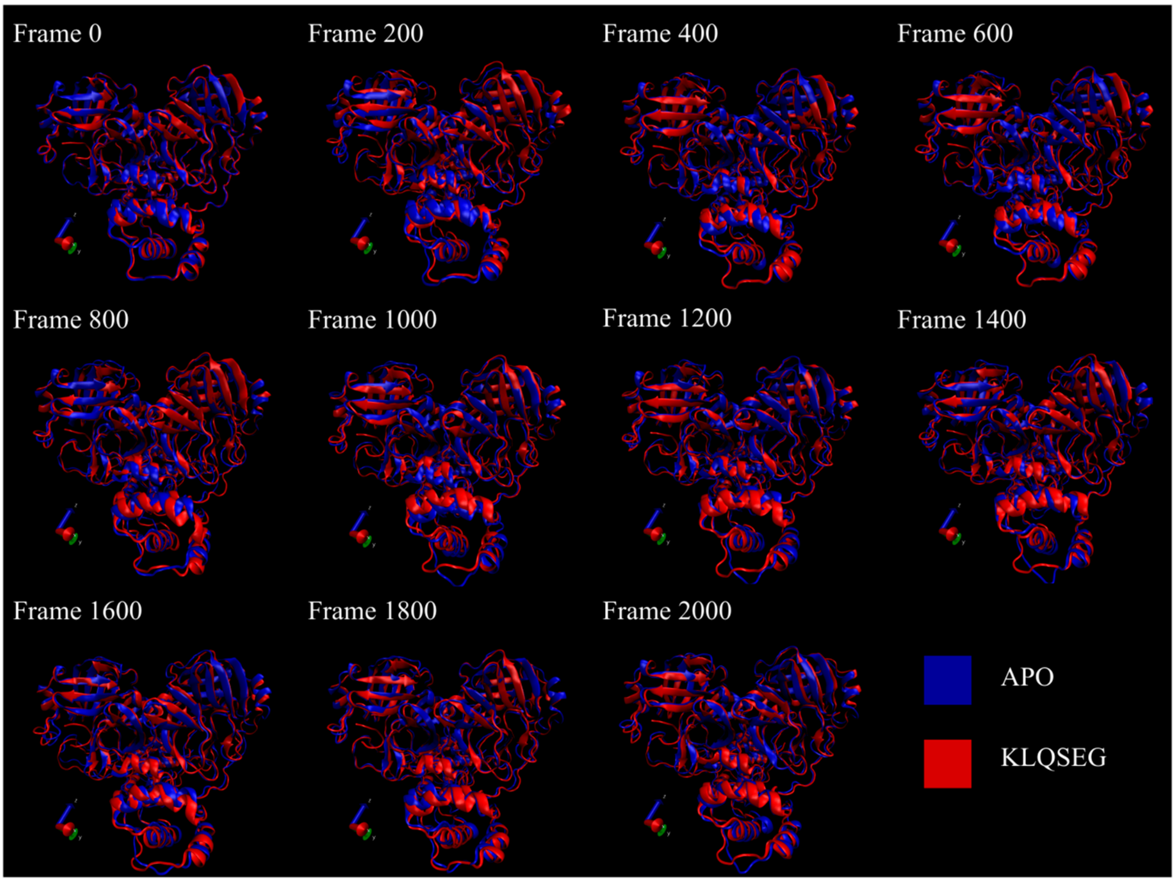

Supplementary Figure S10. Visualisation of the MD trajectories for Group 2 systems. Protein systems are shown cartoon representation, showing *apo*-M^{pro} in blue; and M^{pro}-KLQSEG in red. Images were generated using VMD.

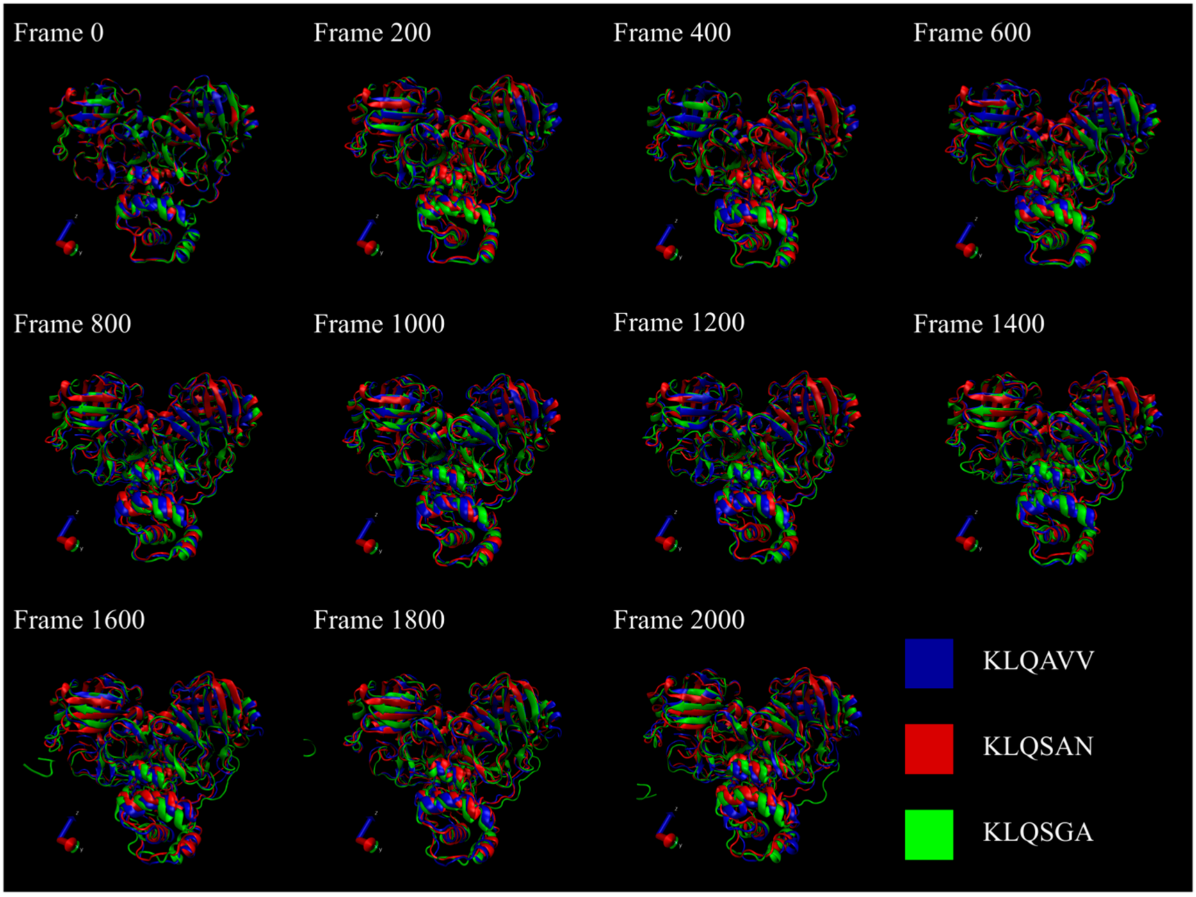

Supplementary Figure S11. Visualisation of the MD trajectories for Group 3 systems. Protein systems are shown cartoon representation, showing M^{pro}-KLQAVV in blue; M^{pro}-KLQSAN in red and M^{pro}-KLQSGA in green. Images were generated using VMD.

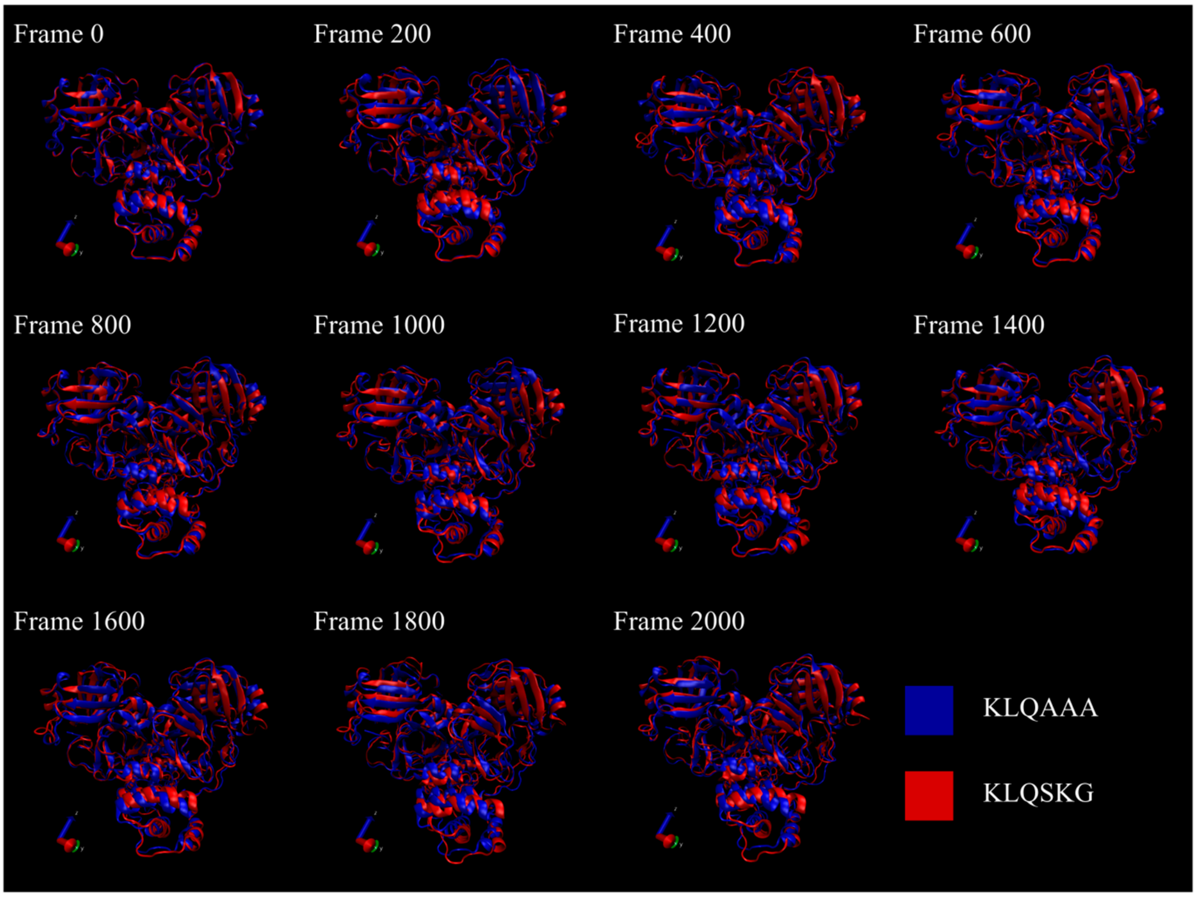

Supplementary Figure S12. Visualisation of the MD trajectories for Group 4 systems. Protein systems are shown cartoon representation, showing M^{pro}-KLQAAA in blue and M^{pro}-KLQSKG in red. Images were generated using VMD.

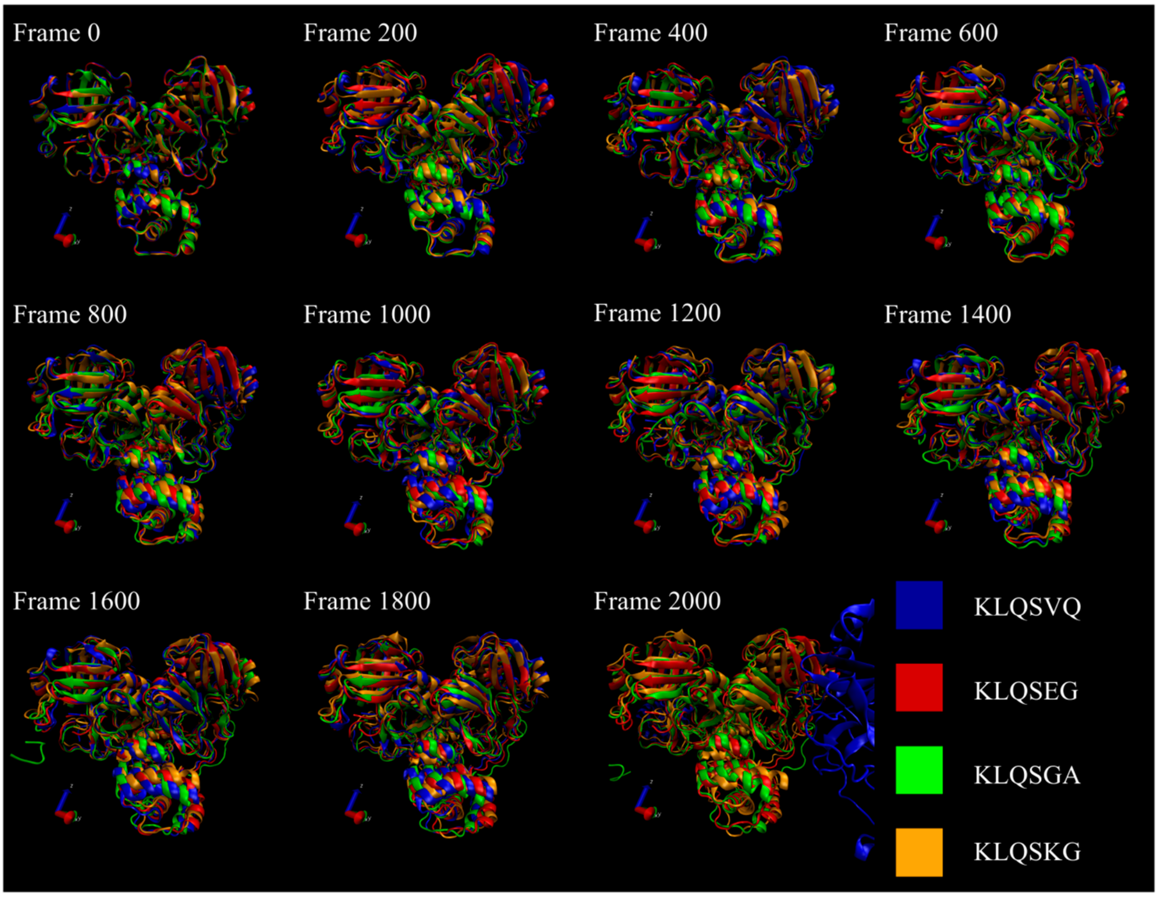

Supplementary Figure S13. Visualisation of the MD trajectories for systems in all hierarchical groups. Protein systems are shown cartoon representation, showing M^{pro}-KLQSVQ (Group 1) in blue; M^{pro}-KLQSEG (Group 2) in red; M^{pro}-KLQSGA (Group 3) in green; and M^{pro}-KLQSKG in orange. Images were generated using VMD.

Supplementary Table S2: RMSD from substrate dynamics ordered by Mean and ordered by Standard Deviation:

RMSD sorted by mean								RMSD sorted by standard deviation						
substrate	mean (nm)	std-dev		substrate	mean (nm)	std-dev		substrate	mean (nm)	std-dev		substrate	mean (nm)	std-dev
KLQAKA	0.169	0.016		KLQANM	0.204	0.028		KLQSAV	0.183	0.016		KLQAAV	0.191	0.026
KLQASG	0.171	0.018		KLQSVA	0.204	0.022		KLQAKA	0.169	0.016		KLQAGA	0.215	0.026
KLQSND	0.172	0.020		KLQSVD	0.204	0.022		KLQAAD	0.180	0.017		KLQSNM	0.179	0.026
KLQSVG	0.173	0.025		KLQSKQ	0.204	0.039		KLQAGG	0.187	0.018		KLQAEE	0.185	0.026
KLQSNQ	0.173	0.019		KLQAEM	0.205	0.020		KLQASG	0.171	0.018		KLQSTQ	0.177	0.027
KLQSSN	0.174	0.019		KLQSSD	0.205	0.023		KLQAAN	0.175	0.018		KLQAED	0.202	0.027
KLQAAN	0.175	0.018		KLQAEG	0.206	0.023		KLQSGV	0.208	0.018		KLQATE	0.207	0.027
KLQAAM	0.177	0.025		APO	0.206	0.023		KLQSSE	0.189	0.019		KLQSTG	0.192	0.027
KLQSAN	0.177	0.023		KLQSKN	0.206	0.028		KLQAKD	0.217	0.019		KLQAAE	0.218	0.027
KLQSTQ	0.177	0.027		KLQSGN	0.206	0.036		KLQSNQ	0.173	0.019		KLQAKM	0.196	0.027
KLQSNM	0.179	0.026		KLQSVV	0.207	0.033		KLQATQ	0.193	0.019		KLQSGE	0.182	0.028
KLQASD	0.179	0.020		KLQSVN	0.207	0.025		KLQSSN	0.174	0.019		KLQANM	0.204	0.028
KLQSGD	0.179	0.023		KLQATE	0.207	0.027		KLQSEE	0.190	0.019		KLQSKN	0.206	0.028
KLQATA	0.180	0.024		KLQSGV	0.208	0.018		KLQSNN	0.193	0.020		KLQAVN	0.216	0.028
KLQAAD	0.180	0.017		KLQAKV	0.209	0.022		KLQSTD	0.203	0.020		KLQSSG	0.216	0.028
KLQSGQ	0.181	0.023		KLQAEV	0.209	0.029		KLQATN	0.197	0.020		KLQSNG	0.228	0.028
KLQATD	0.181	0.021		KLQANQ	0.209	0.025		KLQSND	0.172	0.020		KLQSLD	0.188	0.028
KLQSLE	0.181	0.024		KLQAAG	0.209	0.023		KLQAVD	0.187	0.020		KLQALA	0.224	0.029
KLQSGE	0.182	0.028		KLQALE	0.209	0.025		KLQAEM	0.205	0.020		KLQAEV	0.209	0.029
KLQSAV	0.183	0.016		KLQATM	0.209	0.024		KLQASD	0.179	0.020		KLQAKE	0.198	0.029
KLQSTE	0.183	0.024		KLQSKM	0.211	0.033		KLQAGE	0.184	0.021		KLQASA	0.227	0.030
KLQAGE	0.184	0.021		KLQALD	0.211	0.040		KLQASE	0.192	0.021		KLQAAA	0.192	0.030
KLQAAQ	0.184	0.024		KLQSKV	0.213	0.030		KLQATD	0.181	0.021		KLQALM	0.217	0.030
KLQAEE	0.185	0.026		KLQANE	0.214	0.025		KLQASV	0.186	0.021		KLQSKV	0.213	0.030
KLQASV	0.186	0.021		KLQAKN	0.214	0.036		KLQSVM	0.189	0.021		KLQSEM	0.224	0.030
KLQAGG	0.187	0.018		KLQASN	0.214	0.031		KLQSEG	0.187	0.021		KLQSEN	0.225	0.030

KLQSEG	0.187	0.021	KLQAGA	0.215	0.026	KLQSVD	0.204	0.022	KLQASN	0.214	0.031
KLQSLN	0.187	0.023	KLQANV	0.215	0.023	KLQSVA	0.204	0.022	KLQSSV	0.247	0.031
KLQAVD	0.187	0.020	KLQSSG	0.216	0.028	KLQSAQ	0.201	0.022	KLQAGM	0.193	0.032
KLQSLD	0.188	0.028	KLQAVN	0.216	0.028	KLQSAE	0.195	0.022	KLQSKD	0.222	0.032
KLQSAG	0.188	0.024	KLQALM	0.217	0.030	KLQAKV	0.209	0.022	KLQALG	0.204	0.032
KLQSVM	0.189	0.021	KLQSEQ	0.217	0.033	KLQAKQ	0.194	0.023	KLQSKA	0.222	0.032
KLQSSE	0.189	0.019	KLQSSQ	0.217	0.033	KLQSGD	0.179	0.023	KLQSLV	0.232	0.032
KLQSEE	0.190	0.019	KLQAKD	0.217	0.019	KLQSGQ	0.181	0.023	KLQSAD	0.229	0.033
KLQASM	0.190	0.024	KLQSLG	0.218	0.033	KLQANV	0.215	0.023	KLQSSQ	0.217	0.033
KLQSVE	0.190	0.025	KLQAAE	0.218	0.027	KLQSLN	0.187	0.023	KLQSLG	0.218	0.033
KLQAAV	0.191	0.026	KLQANA	0.218	0.050	APO	0.206	0.023	KLQSEQ	0.217	0.033
KLQAAA	0.192	0.030	KLQSKA	0.222	0.032	KLQAAG	0.209	0.023	KLQATV	0.226	0.033
KLQASE	0.192	0.021	KLQSEA	0.222	0.041	KLQAKG	0.194	0.023	KLQSTA	0.242	0.033
KLQAGQ	0.192	0.025	KLQSKD	0.222	0.032	KLQAEG	0.206	0.023	KLQSKM	0.211	0.033
KLQSTG	0.192	0.027	KLQALV	0.223	0.024	KLQALN	0.224	0.023	KLQSVV	0.207	0.033
KLQSTM	0.193	0.040	KLQALN	0.224	0.023	KLQSAN	0.177	0.023	KLQSAM	0.230	0.035
KLQAGM	0.193	0.032	KLQSEM	0.224	0.030	KLQSSD	0.205	0.023	KLQSGN	0.206	0.036
KLQSNN	0.193	0.020	KLQALA	0.224	0.029	KLQASM	0.190	0.024	KLQAEA	0.194	0.036
KLQATQ	0.193	0.019	KLQSEN	0.225	0.030	KLQALV	0.223	0.024	KLQAKN	0.214	0.036
KLQAKQ	0.194	0.023	KLQATV	0.226	0.033	KLQATM	0.209	0.024	KLQSNA	0.226	0.036
KLQAVM	0.194	0.024	KLQSNA	0.226	0.036	KLQAVM	0.194	0.024	KLQSTN	0.244	0.037
KLQAEA	0.194	0.036	KLQASA	0.227	0.030	KLQANG	0.227	0.024	KLQSLQ	0.233	0.037
KLQAKG	0.194	0.023	KLQANG	0.227	0.024	KLQSAG	0.188	0.024	KLQSKQ	0.204	0.039
KLQSLM	0.194	0.043	KLQSNG	0.228	0.028	KLQSLE	0.181	0.024	KLQALD	0.211	0.040
KLQSAE	0.195	0.022	KLQSAD	0.229	0.033	KLQALQ	0.202	0.024	KLQAGD	0.244	0.040
KLQAKM	0.196	0.027	KLQSAM	0.230	0.035	KLQAVV	0.200	0.024	KLQSTM	0.193	0.040
KLQSKG	0.196	0.051	KLQSLV	0.232	0.032	KLQAAQ	0.184	0.024	KLQSAA	0.203	0.041
KLQATN	0.197	0.020	KLQSLQ	0.233	0.037	KLQASQ	0.199	0.024	KLQSEA	0.222	0.041
KLQAKE	0.198	0.029	KLQSGA	0.238	0.187	KLQSTE	0.183	0.024	KLQAVQ	0.240	0.042
KLQASQ	0.199	0.024	KLQAVQ	0.240	0.042	KLQATA	0.180	0.024	KLQAVA	0.267	0.043
KLQAVV	0.200	0.024	KLQSTA	0.242	0.033	KLQAGQ	0.192	0.025	KLQSLM	0.194	0.043
KLQATG	0.200	0.025	KLQAGD	0.244	0.040	KLQSVE	0.190	0.025	KLQAGV	0.259	0.049

KLQSAQ	0.201	0.022
KLQAEQ	0.201	0.050
KLQALQ	0.202	0.024
KLQAED	0.202	0.027
KLQSTD	0.203	0.020
KLQSAA	0.203	0.041
KLQAVG	0.203	0.025
KLQALG	0.204	0.032

KLQSTN	0.244	0.037
KLQAVE	0.245	0.063
KLQSSV	0.247	0.031
KLQSLA	0.251	0.057
KLQAGV	0.259	0.049
KLQAVA	0.267	0.043
KLQSVQ	0.271	0.564
KLQAND	0.320	0.636

KLQALE	0.209	0.025
KLQSVN	0.207	0.025
KLQAVG	0.203	0.025
KLQSVG	0.173	0.025
KLQANE	0.214	0.025
KLQAAM	0.177	0.025
KLQATG	0.200	0.025
KLQANQ	0.209	0.025

KLQAEQ	0.201	0.050
KLQANA	0.218	0.050
KLQSKG	0.196	0.051
KLQSLA	0.251	0.057
KLQAVE	0.245	0.063
KLQSGA	0.238	0.187
KLQSVQ	0.271	0.564
KLQAND	0.320	0.636

Supplementary Table S3: Rg from substrate dynamics ordered by Mean and ordered by Standard Deviation:

Rg sorted by mean						
substrate	mean (nm)	std-dev		substrate	mean (nm)	std-dev
KLQSAM	2.5704	0.0146		KLQALD	2.5923	0.0082
KLQAAD	2.5740	0.0125		APO	2.5924	0.0089
KLQANG	2.5743	0.0095		KLQAVV	2.5925	0.0084
KLQSEA	2.5749	0.0127		KLQSVM	2.5925	0.0083
KLQALV	2.5752	0.0102		KLQASE	2.5925	0.0078
KLQSLA	2.5760	0.0154		KLQSVE	2.5927	0.0078
KLQSEN	2.5764	0.0104		KLQSKV	2.5928	0.0074
KLQSSN	2.5788	0.0102		KLQASM	2.5928	0.0090
KLQAKN	2.5798	0.0118		KLQSTN	2.5930	0.0091
KLQAGQ	2.5804	0.0083		KLQAAQ	2.5934	0.0079
KLQAKE	2.5812	0.0093		KLQAKA	2.5934	0.0086
KLQATG	2.5813	0.0101		KLQATD	2.5935	0.0082
KLQSLE	2.5817	0.0101		KLQSSV	2.5936	0.0116
KLQAGD	2.5821	0.0110		KLQSNN	2.5936	0.0075
KLQAGM	2.5821	0.0112		KLQSLV	2.5936	0.0102
KLQANE	2.5826	0.0091		KLQAKD	2.5937	0.0091
KLQAKQ	2.5828	0.0117		KLQAEG	2.5938	0.0089
KLQALQ	2.5830	0.0092		KLQSGN	2.5939	0.0094
KLQSEM	2.5831	0.0084		KLQSVD	2.5940	0.0073
KLQANM	2.5839	0.0087		KLQAAA	2.5940	0.0121
KLQATN	2.5842	0.0132		KLQSGV	2.5951	0.0087
KLQSNA	2.5842	0.0110		KLQSNG	2.5953	0.0082
KLQSAD	2.5845	0.0087		KLQSSD	2.5953	0.0101
KLQSVN	2.5845	0.0103		KLQSKM	2.5954	0.0127
KLQAVM	2.5845	0.0116		KLQSKQ	2.5955	0.0093
KLQSKA	2.5845	0.0083		KLQATA	2.5955	0.0109

Rg sorted by standard deviation						
substrate	mean (nm)	std-dev		substrate	mean (nm)	std-dev
KLQAAG	2.5911	0.0072		KLQAAM	2.5885	0.0093
KLQAGG	2.5856	0.0072		KLQAVN	2.5898	0.0093
KLQSVD	2.5940	0.0073		KLQSAV	2.5913	0.0094
KLQSVV	2.5995	0.0073		KLQSKG	2.5973	0.0094
KLQSKV	2.5928	0.0074		KLQSGN	2.5939	0.0094
KLQAGA	2.5895	0.0074		KLQAEM	2.5885	0.0094
KLQSND	2.5962	0.0074		KLQSTD	2.5852	0.0094
KLQSNN	2.5936	0.0075		KLQALM	2.5987	0.0094
KLQAKG	2.5905	0.0075		KLQANG	2.5743	0.0095
KLQAKV	2.5905	0.0076		KLQSLD	2.5905	0.0095
KLQAVG	2.6046	0.0077		KLQAEA	2.5895	0.0096
KLQASE	2.5925	0.0078		KLQATV	2.5854	0.0097
KLQSVE	2.5927	0.0078		KLQSLQ	2.5956	0.0099
KLQATQ	2.5973	0.0078		KLQATG	2.5813	0.0101
KLQSGD	2.6039	0.0078		KLQSLE	2.5817	0.0101
KLQAAQ	2.5934	0.0079		KLQSSD	2.5953	0.0101
KLQSAG	2.6001	0.0080		KLQSLV	2.5936	0.0102
KLQASG	2.6034	0.0080		KLQSTE	2.5915	0.0102
KLQSEE	2.5984	0.0080		KLQSSN	2.5788	0.0102
KLQSGE	2.6007	0.0081		KLQAKM	2.6036	0.0102
KLQSNG	2.5953	0.0082		KLQALV	2.5752	0.0102
KLQAED	2.5889	0.0082		KLQSVN	2.5845	0.0103
KLQATD	2.5935	0.0082		KLQSNQ	2.5958	0.0103
KLQALG	2.6041	0.0082		KLQASA	2.5979	0.0103
KLQALD	2.5923	0.0082		KLQSEN	2.5764	0.0104
KLQAGQ	2.5804	0.0083		KLQANA	2.5986	0.0105

KLQALE	2.5847	0.0129	KLQSLQ	2.5956	0.0099	KLQSKA	2.5845	0.0083	KLQAEV	2.5884	0.0105
KLQANV	2.5847	0.0086	KLQSAN	2.5958	0.0084	KLQSVM	2.5925	0.0083	KLQSLG	2.5871	0.0105
KLQALN	2.5848	0.0083	KLQSNQ	2.5958	0.0103	KLQALN	2.5848	0.0083	KLQAGV	2.5915	0.0107
KLQSTD	2.5852	0.0094	KLQSND	2.5962	0.0074	KLQSSE	2.6050	0.0083	KLQAEQ	2.6043	0.0108
KLQATV	2.5854	0.0097	KLQSNM	2.5963	0.0130	KLQAVV	2.5925	0.0084	KLQATA	2.5955	0.0109
KLQAGG	2.5856	0.0072	KLQSKD	2.5967	0.0118	KLQSTG	2.5865	0.0084	KLQSEG	2.5993	0.0110
KLQATM	2.5860	0.0090	KLQASQ	2.5968	0.0085	KLQANQ	2.5991	0.0084	KLQAGD	2.5821	0.0110
KLQAVE	2.5862	0.0125	KLQSVG	2.5971	0.0125	KLQSAN	2.5958	0.0084	KLQSNA	2.5842	0.0110
KLQSTG	2.5865	0.0084	KLQSKG	2.5973	0.0094	KLQSEM	2.5831	0.0084	KLQSTQ	2.5976	0.0112
KLQAEE	2.5867	0.0087	KLQATQ	2.5973	0.0078	KLQASQ	2.5968	0.0085	KLQAGM	2.5821	0.0112
KLQSLG	2.5871	0.0105	KLQSTQ	2.5976	0.0112	KLQSAA	2.5913	0.0085	KLQSLM	2.5884	0.0112
KLQSAE	2.5876	0.0117	KLQASA	2.5979	0.0103	KLQASD	2.5918	0.0085	KLQSLN	2.6006	0.0112
KLQSLM	2.5884	0.0112	KLQSEE	2.5984	0.0080	KLQAKA	2.5934	0.0086	KLQSSQ	2.6017	0.0113
KLQAEV	2.5884	0.0105	KLQANA	2.5986	0.0105	KLQANV	2.5847	0.0086	KLQAVM	2.5845	0.0116
KLQSVA	2.5885	0.0091	KLQALM	2.5987	0.0094	KLQSGQ	2.6045	0.0086	KLQSKN	2.5889	0.0116
KLQAAM	2.5885	0.0093	KLQANQ	2.5991	0.0084	KLQAVD	2.5999	0.0086	KLQSSV	2.5936	0.0116
KLQAEM	2.5885	0.0094	KLQSEG	2.5993	0.0110	KLQALA	2.5903	0.0086	KLQAKQ	2.5828	0.0117
KLQAGE	2.5887	0.0093	KLQSVV	2.5995	0.0073	KLQSAD	2.5845	0.0087	KLQSAE	2.5876	0.0117
KLQSKN	2.5889	0.0116	KLQAVD	2.5999	0.0086	KLQSSG	2.6106	0.0087	KLQAAV	2.6148	0.0118
KLQAED	2.5889	0.0082	KLQSAG	2.6001	0.0080	KLQANM	2.5839	0.0087	KLQAKN	2.5798	0.0118
KLQAVQ	2.5891	0.0122	KLQSTA	2.6005	0.0092	KLQAEE	2.5867	0.0087	KLQSKD	2.5967	0.0118
KLQAGA	2.5895	0.0074	KLQSLN	2.6006	0.0112	KLQSGV	2.5951	0.0087	KLQAAA	2.5940	0.0121
KLQAEA	2.5895	0.0096	KLQSGE	2.6007	0.0081	KLQAAN	2.6061	0.0088	KLQAVQ	2.5891	0.0122
KLQAAE	2.5898	0.0174	KLQSSQ	2.6017	0.0113	KLQSAQ	2.5907	0.0088	KLQAAD	2.5740	0.0125
KLQAVN	2.5898	0.0093	KLQASG	2.6034	0.0080	KLQAVA	2.6272	0.0088	KLQAVE	2.5862	0.0125
KLQALA	2.5903	0.0086	KLQAKM	2.6036	0.0102	APO	2.5924	0.0089	KLQSVG	2.5971	0.0125
KLQASV	2.5903	0.0138	KLQSGD	2.6039	0.0078	KLQAEG	2.5938	0.0089	KLQSEA	2.5749	0.0127
KLQSLD	2.5905	0.0095	KLQALG	2.6041	0.0082	KLQASN	2.5909	0.0090	KLQSKM	2.5954	0.0127
KLQAKG	2.5905	0.0075	KLQAEQ	2.6043	0.0108	KLQASM	2.5928	0.0090	KLQSEQ	2.6187	0.0128
KLQAKV	2.5905	0.0076	KLQSGQ	2.6045	0.0086	KLQATM	2.5860	0.0090	KLQALE	2.5847	0.0129
KLQSAQ	2.5907	0.0088	KLQAVG	2.6046	0.0077	KLQSTM	2.5914	0.0090	KLQSNM	2.5963	0.0130
KLQASN	2.5909	0.0090	KLQSSE	2.6050	0.0083	KLQANE	2.5826	0.0091	KLQATN	2.5842	0.0132

KLQAAG	2.5911	0.0072	KLQAAN	2.6061	0.0088	KLQSTN	2.5930	0.0091	KLQASV	2.5903	0.0138
KLQSAV	2.5913	0.0094	KLQSGA	2.6071	0.0256	KLQSVA	2.5885	0.0091	KLQSAM	2.5704	0.0146
KLQSAA	2.5913	0.0085	KLQSSG	2.6106	0.0087	KLQAKD	2.5937	0.0091	KLQATE	2.5917	0.0146
KLQSTM	2.5914	0.0090	KLQAAV	2.6148	0.0118	KLQSTA	2.6005	0.0092	KLQSLA	2.5760	0.0154
KLQAGV	2.5915	0.0107	KLQSEQ	2.6187	0.0128	KLQALQ	2.5830	0.0092	KLQAAE	2.5898	0.0174
KLQSTE	2.5915	0.0102	KLQAVA	2.6272	0.0088	KLQAGE	2.5887	0.0093	KLQSGA	2.6071	0.0256
KLQATE	2.5917	0.0146	KLQSVQ	2.6596	0.3865	KLQAKE	2.5812	0.0093	KLQSVQ	2.6596	0.3865
KLQASD	2.5918	0.0085	KLQAND	2.6605	0.4222	KLQSKQ	2.5955	0.0093	KLQAND	2.6605	0.4222

Supplementary Table S4: RMSD and Rg from substrate dynamics ordered by substrate

RMSD sorted by residue

substrate	mean (nm)	std-dev		substrate	mean (nm)	std-dev
APO	0.206	0.023		KLQAVM	0.194	0.024
KLQAAA	0.192	0.030		KLQAVN	0.216	0.028
KLQAAD	0.180	0.017		KLQAVQ	0.240	0.042
KLQAAE	0.218	0.027		KLQAVV	0.200	0.024
KLQAAG	0.209	0.023		KLQSAA	0.203	0.041
KLQAAM	0.177	0.025		KLQSAD	0.229	0.033
KLQAAN	0.175	0.018		KLQSAE	0.195	0.022
KLQAAQ	0.184	0.024		KLQSAG	0.188	0.024
KLQAAV	0.191	0.026		KLQSAM	0.230	0.035
KLQAEA	0.194	0.036		KLQSAN	0.177	0.023
KLQAED	0.202	0.027		KLQSAQ	0.201	0.022
KLQAEE	0.185	0.026		KLQSAV	0.183	0.016
KLQAEG	0.206	0.023		KLQSEA	0.222	0.041
KLQAEM	0.205	0.020		KLQSEE	0.190	0.019
KLQAEQ	0.201	0.050		KLQSEG	0.187	0.021
KLQAEV	0.209	0.029		KLQSEM	0.224	0.030
KLQAGA	0.215	0.026		KLQSEN	0.225	0.030
KLQAGD	0.244	0.040		KLQSEQ	0.217	0.033
KLQAGE	0.184	0.021		KLQSGA	0.238	0.187
KLQAGG	0.187	0.018		KLQSGD	0.179	0.023
KLQAGM	0.193	0.032		KLQSGE	0.182	0.028
KLQAGQ	0.192	0.025		KLQSGN	0.206	0.036
KLQAGV	0.259	0.049		KLQSGQ	0.181	0.023
KLQAKA	0.169	0.016		KLQSGV	0.208	0.018
KLQAKD	0.217	0.019		KLQSKA	0.222	0.032
KLQAKE	0.198	0.029		KLQSKD	0.222	0.032

Rg sorted by residue

substrate	mean (nm)	std-dev		substrate	mean (nm)	std-dev
APO	2.5924	0.0089		KLQAVM	2.5845	0.0116
KLQAAA	2.5940	0.0121		KLQAVN	2.5898	0.0093
KLQAAD	2.5740	0.0125		KLQAVQ	2.5891	0.0122
KLQAAE	2.5898	0.0174		KLQAVV	2.5925	0.0084
KLQAAG	2.5911	0.0072		KLQSAA	2.5913	0.0085
KLQAAM	2.5885	0.0093		KLQSAD	2.5845	0.0087
KLQAAN	2.6061	0.0088		KLQSAE	2.5876	0.0117
KLQAAQ	2.5934	0.0079		KLQSAG	2.6001	0.0080
KLQAAV	2.6148	0.0118		KLQSAM	2.5704	0.0146
KLQAEA	2.5895	0.0096		KLQSAN	2.5958	0.0084
KLQAED	2.5889	0.0082		KLQSAQ	2.5907	0.0088
KLQAEE	2.5867	0.0087		KLQSAV	2.5913	0.0094
KLQAEG	2.5938	0.0089		KLQSEA	2.5749	0.0127
KLQAEM	2.5885	0.0094		KLQSEE	2.5984	0.0080
KLQAEQ	2.6043	0.0108		KLQSEG	2.5993	0.0110
KLQAEV	2.5884	0.0105		KLQSEM	2.5831	0.0084
KLQAGA	2.5895	0.0074		KLQSEN	2.5764	0.0104
KLQAGD	2.5821	0.0110		KLQSEQ	2.6187	0.0128
KLQAGE	2.5887	0.0093		KLQSGA	2.6071	0.0256
KLQAGG	2.5856	0.0072		KLQSGD	2.6039	0.0078
KLQAGM	2.5821	0.0112		KLQSGE	2.6007	0.0081
KLQAGQ	2.5804	0.0083		KLQSGN	2.5939	0.0094
KLQAGV	2.5915	0.0107		KLQSGQ	2.6045	0.0086
KLQAKA	2.5934	0.0086		KLQSGV	2.5951	0.0087
KLQAKD	2.5937	0.0091		KLQSKA	2.5845	0.0083
KLQAKE	2.5812	0.0093		KLQSKD	2.5967	0.0118

KLQAKG	0.194	0.023	KLQSKG	0.196	0.051	KLQAKG	2.5905	0.0075	KLQSKG	2.5973	0.0094
KLQAKM	0.196	0.027	KLQSKM	0.211	0.033	KLQAKM	2.6036	0.0102	KLQSKM	2.5954	0.0127
KLQAKN	0.214	0.036	KLQSKN	0.206	0.028	KLQAKN	2.5798	0.0118	KLQSKN	2.5889	0.0116
KLQAKQ	0.194	0.023	KLQSKQ	0.204	0.039	KLQAKQ	2.5828	0.0117	KLQSKQ	2.5955	0.0093
KLQAKV	0.209	0.022	KLQSKV	0.213	0.030	KLQAKV	2.5905	0.0076	KLQSKV	2.5928	0.0074
KLQALA	0.224	0.029	KLQSLA	0.251	0.057	KLQALA	2.5903	0.0086	KLQSLA	2.5760	0.0154
KLQALD	0.211	0.040	KLQSLD	0.188	0.028	KLQALD	2.5923	0.0082	KLQSLD	2.5905	0.0095
KLQALE	0.209	0.025	KLQSLE	0.181	0.024	KLQALE	2.5847	0.0129	KLQSLE	2.5817	0.0101
KLQALG	0.204	0.032	KLQSLG	0.218	0.033	KLQALG	2.6041	0.0082	KLQSLG	2.5871	0.0105
KLQALM	0.217	0.030	KLQSLM	0.194	0.043	KLQALM	2.5987	0.0094	KLQSLM	2.5884	0.0112
KLQALN	0.224	0.023	KLQSLN	0.187	0.023	KLQALN	2.5848	0.0083	KLQSLN	2.6006	0.0112
KLQALQ	0.202	0.024	KLQSLQ	0.233	0.037	KLQALQ	2.5830	0.0092	KLQSLQ	2.5956	0.0099
KLQALV	0.223	0.024	KLQSLV	0.232	0.032	KLQALV	2.5752	0.0102	KLQSLV	2.5936	0.0102
KLQANA	0.218	0.050	KLQSNA	0.226	0.036	KLQANA	2.5986	0.0105	KLQSNA	2.5842	0.0110
KLQAND	0.320	0.636	KLQSND	0.172	0.020	KLQAND	2.6605	0.4222	KLQSND	2.5962	0.0074
KLQANE	0.214	0.025	KLQSNG	0.228	0.028	KLQANE	2.5826	0.0091	KLQSNG	2.5953	0.0082
KLQANG	0.227	0.024	KLQSNM	0.179	0.026	KLQANG	2.5743	0.0095	KLQSNM	2.5963	0.0130
KLQANM	0.204	0.028	KLQSNN	0.193	0.020	KLQANM	2.5839	0.0087	KLQSNN	2.5936	0.0075
KLQANQ	0.209	0.025	KLQSNQ	0.173	0.019	KLQANQ	2.5991	0.0084	KLQSNQ	2.5958	0.0103
KLQANV	0.215	0.023	KLQSSD	0.205	0.023	KLQANV	2.5847	0.0086	KLQSSD	2.5953	0.0101
KLQASA	0.227	0.030	KLQSSE	0.189	0.019	KLQASA	2.5979	0.0103	KLQSSE	2.6050	0.0083
KLQASD	0.179	0.020	KLQSSG	0.216	0.028	KLQASD	2.5918	0.0085	KLQSSG	2.6106	0.0087
KLQASE	0.192	0.021	KLQSSN	0.174	0.019	KLQASE	2.5925	0.0078	KLQSSN	2.5788	0.0102
KLQASG	0.171	0.018	KLQSSQ	0.217	0.033	KLQASG	2.6034	0.0080	KLQSSQ	2.6017	0.0113
KLQASM	0.190	0.024	KLQSSV	0.247	0.031	KLQASM	2.5928	0.0090	KLQSSV	2.5936	0.0116
KLQASN	0.214	0.031	KLQSTA	0.242	0.033	KLQASN	2.5909	0.0090	KLQSTA	2.6005	0.0092
KLQASQ	0.199	0.024	KLQSTD	0.203	0.020	KLQASQ	2.5968	0.0085	KLQSTD	2.5852	0.0094
KLQASV	0.186	0.021	KLQSTE	0.183	0.024	KLQASV	2.5903	0.0138	KLQSTE	2.5915	0.0102
KLQATA	0.180	0.024	KLQSTG	0.192	0.027	KLQATA	2.5955	0.0109	KLQSTG	2.5865	0.0084
KLQATD	0.181	0.021	KLQSTM	0.193	0.040	KLQATD	2.5935	0.0082	KLQSTM	2.5914	0.0090
KLQATE	0.207	0.027	KLQSTN	0.244	0.037	KLQATE	2.5917	0.0146	KLQSTN	2.5930	0.0091

KLQATG	0.200	0.025
KLQATM	0.209	0.024
KLQATN	0.197	0.020
KLQATQ	0.193	0.019
KLQATV	0.226	0.033
KLQAVA	0.267	0.043
KLQAVD	0.187	0.020
KLQAVE	0.245	0.063
KLQAVG	0.203	0.025

KLQSTQ	0.177	0.027
KLQSVA	0.204	0.022
KLQSVD	0.204	0.022
KLQSVE	0.190	0.025
KLQSVG	0.173	0.025
KLQSVM	0.189	0.021
KLQSVN	0.207	0.025
KLQSVQ	0.271	0.564
KLQSVV	0.207	0.033

KLQATG	2.5813	0.0101
KLQATM	2.5860	0.0090
KLQATN	2.5842	0.0132
KLQATQ	2.5973	0.0078
KLQATV	2.5854	0.0097
KLQAVA	2.6272	0.0088
KLQAVD	2.5999	0.0086
KLQAVE	2.5862	0.0125
KLQAVG	2.6046	0.0077

KLQSTQ	2.5976	0.0112
KLQSVA	2.5885	0.0091
KLQSVD	2.5940	0.0073
KLQSVE	2.5927	0.0078
KLQSVG	2.5971	0.0125
KLQSVM	2.5925	0.0083
KLQSVN	2.5845	0.0103
KLQSVQ	2.6596	0.3865
KLQSVV	2.5995	0.0073

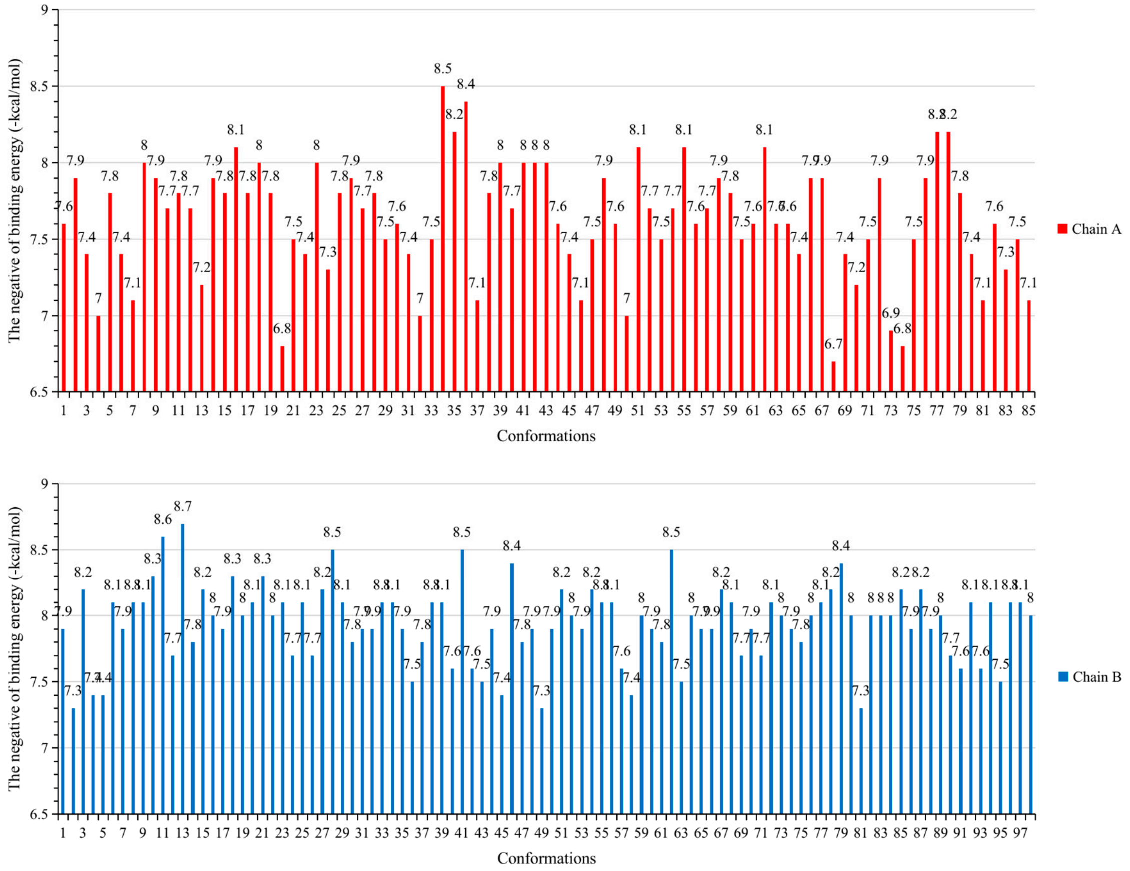

Supplementary Figure S14. Preliminary docking studies to determine the protein chain to prioritise for docking studies. The negative of the docking scores of the Arg-Leu-Gln-Ala-Ala-Asn (RLQAAN) conformers were plotted on bar graphs, showing the docking scores of chain A (red) and chain B (blue). The image was generated using WPS Spreadsheet 2019.
